# Synthesis and Characterization of Stimuli-Responsive Polymer Brushes in Nanofluidic Channels

**DOI:** 10.1021/acsami.3c12744

**Published:** 2023-11-16

**Authors:** Hadi Rahmaninejad, Andrew J. Parnell, Wei-Liang Chen, Nilay Duzen, Thomas Sexton, Gary Dunderdale, John F. Ankner, Wim Bras, Christopher K. Ober, Anthony J. Ryan, Rana Ashkar

**Affiliations:** † Department of Physics, Virginia Tech, Blacksburg, Virginia 24061, United States; ‡ Center for Soft Matter and Biological Physics, Virginia Tech, Blacksburg, Virginia 24061, United States; § Department of Physics, The University of Sheffield, Sheffield S3 7RH, U.K.; ∥ Department of Material Science and Engineering, University of Pennsylvania, Philadelphia, Pennsylvania 19104, United States; ⊥ Department of Material Science and Engineering, Cornell University, Ithaca, New York 14850, United States; # Department of Chemical and Biological Engineering, The University of Sheffield, Sheffield S1 3JD, U.K.; ¶ Second Target Station, Oak Ridge National Laboratory, Oak Ridge, Tennessee 37830, United States; ∇ Chemical Sciences Division, Oak Ridge National Laboratory, Oak Ridge, Tennessee 37830, United States; ○ Department of Chemistry, The University of Sheffield, Sheffield S3 7HF, U.K.; ⧫ Department of Physics, Virginia Tech, Blacksburg, Virginia 24061, United States; †† Macromolecular Innovation Institute, Virginia Tech, Blacksburg, Virginia 24061, United States

**Keywords:** polyelectrolyte polymers, pH-response, brush conformations, neutron reflectometry, off-specular scattering, dynamical theory analysis

## Abstract

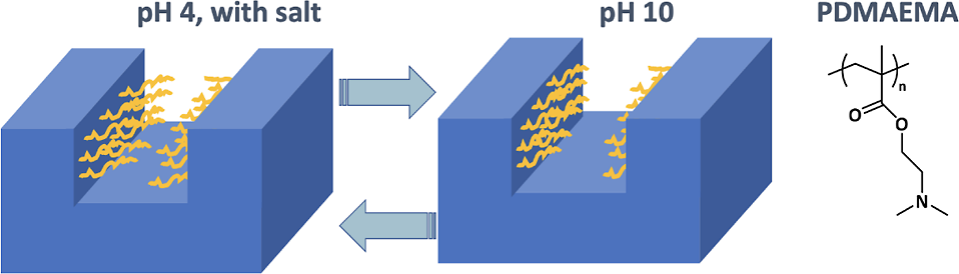

Nanochannels with controllable gating behavior are attractive features in a wide range of nanofluidic applications including viral detection, particle sorting, and flow regulation. Here, we use selective sidewall functionalization of nanochannels with a polyelectrolyte brush to investigate the channel gating response to variations in solution pH and ionic strength. The conformational and structural changes of the interfacial brush layer within the channels are interrogated by specular and off-specular neutron reflectometry. Simultaneous fits of the specular and off-specular signals, using a dynamical theory model and a fitting optimization protocol, enable detailed characterization of the brush conformations and corresponding channel geometry under different solution conditions. Our results indicate a collapsed brush state under basic pH, equivalent to an open gate, and an expanded brush state representing a partially closed gate upon decreasing the pH and salt concentration. These findings open new possibilities in noninvasive in situ characterization of tunable nanofluidics and lab-on-chip devices with advanced designs and improved functionality.

## 1 Introduction

Nanofluidics with controllable gating properties have promising and exciting uses in many research areas requiring regulated fluid flow and particle selectivity, including fluidic diodes,^1^ molecular sorting,^2^ drug delivery,^3^ cell identification,^4^ DNA analysis,^5−7^ and water purification.^8^ Various designs have been proposed and examined for the controlled gating behavior in nanochannels, such as the use of surface charge or electric fields for direct ionic manipulation in channels that are dimensionally comparable to the Debye length.^9^ However, approaches based on the physical manipulation of nanochannel geometry pose a difficult challenge for practical devices with tunable or responsive gating properties. Fortunately, advances in nanochannel functionalization with stimuli-responsive polymers adequately address these challenges and enable new opportunities for advanced applications requiring flow regulation on the nanoscale.^10−13^ The conformational changes of stimuli-responsive polymers with thermal, chemical, or optical variations in the immediate environment present a versatile means for designing smart functional surfaces or interfaces.^14,15^ Indeed, the ability of certain polymers to tune surface properties such as adhesion, roughness, wettability,^16−18^ reactivity, and selectivity has facilitated novel designs of devices with controlled flow and transport.^19−21^ Recently, stimuli-responsive polymers have also attracted significant attention in biotechnology and biomedical applications.^15^ Polyelectrolyte polymers, in particular, are commonly used in applications where variations in pH and ionic strength can be utilized for drug delivery,^22^ separation processes,^23^ tissue engineering,^24^ and biosensors or actuators.^25^

In this study, we use poly(dimethyl aminoethyl methacrylate) (PDMAEMA), a pH-responsive polymer, to examine the potential of polyelectrolytes in the design of configurable nanochannels. PDMAEMA is characterized by amine pendant groups (basic groups)^26^ which get protonated in acidic solutions, resulting in a swelling of the polymer due to the osmotic effect of counterions. More importantly, PDMAEMA brushes show sharp and reversible switching behavior between the swollen and collapsed state,^27^ an attractive property for tunable nanofluidics.^28^ To examine this possibility, we employed selective side-wall functionalization of submicron silicon channels with PDMAEMA brushes and tested the resultant channel geometry under different solution conditions. Specifically, we explored the degree of brush swelling and collapse in response to variation in the solution pH and salt concentration. The interdependence of the brush conformation and effective channel width has significant implications in controlled gating and nanoparticle sorting applications.

A full 3D characterization of the brush-decorated channels was obtained using specular and off-specular neutron reflectometry (NR). The measurements were performed on a series of periodic linear channels. This multichannel geometry is necessary for signal amplification and subsequent data analysis. All measurements were performed in a fluid cell, with microfluidic ports, that was custom-designed for neutron reflectometry studies.^29^ To maximize the neutron scattering contrast between the polymer brushes and their aqueous environment, all NR measurements were performed in D_2_O buffers with the desired pH or salt content. This solvent deuteration scheme yields the highest achievable contrast between the polymer and the buffer.^30−32^ Here, we note that the scattering geometry in our NR measurements significantly differs from specular reflectometry studies on polymer brushes grafted onto flat surfaces.^27,33,34^ In such experiments, the scattering signal is primarily specular and is typically analyzed using standard reflectometry models available in NR data fitting packages.

**Figure 1:**
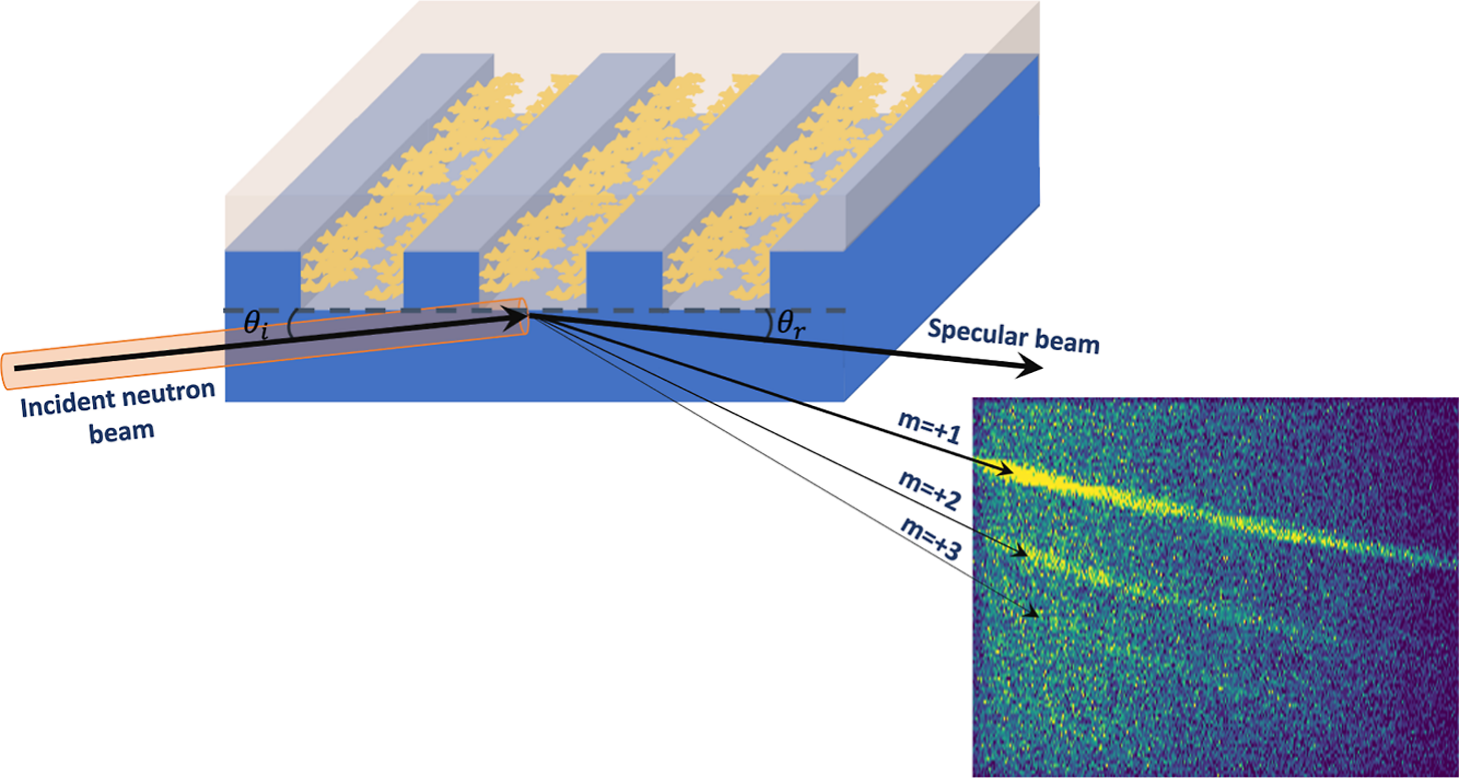
Schematic of neutron reflectometry on periodic brush-functionalized nanochannels. Figure showing the scattering geometry of the specular signal as well as the off-specular (or Bragg) signal resulting from channel periodicity. In off-specular measurements, the specular signal is masked to enable better signal-to-noise detection of the less intense Bragg signals, as shown in the embedded detector image.

In contrast, the channel periodicity in the present study results in intense off-specular scattering (besides the specular signal; see Figure 1) that cannot be analyzed by standard fitting protocols. Instead, we adapted a dynamical scattering theory model, previously developed and validated by Ashkar et al.,^35^ which provides an exact solution for scattering from periodic structures. Our approach provides the ability to perform simultaneous fitting of specular and off-specular signals, overcoming the well-known phase problem in the analysis of NR data.^36^ This combined specular/off-specular fitting approach yielded unique fits of the 3D structure of the polymer brushes within the channels. The data fits, performed using a computational optimization protocol, indicate that the polymer chains assume a collapsed state under basic pH conditions, resulting in an open gate structure. However, upon decreasing the solution pH, the brushes swell and cause a decrease in the gate width. As the salt concentration increases, the brushes initially experience further swelling before slightly collapsing at high salt concentrations. Interestingly, our studies suggests that in the swollen brush state, there is a dense polymer brush region near the channel walls and a less dense region toward the middle of the channels, consistent with previous studies of polymer brushes on flat substrates.^26,27,37,38^

## 2 Results and Discussion

NR measurements were performed on periodic channels decorated with PDMAEMA brushes, with a dry thickness of 94.6 nm and a grafting density of 0.7 chains/nm^2^. The measurements were conducted under various solution conditions, including different pH values (pH 4 and pH 10) and salt concentrations (10, 100, and 1000 mM). The fabricated channels had a width of ∼500 nm and a repeat distance of ∼1 μm. These measurements were conducted in specular and off-specular scattering geometries with the incident neutron beam impinging on the channels from the silicon substrate. Specular signals primarily yielded the in-depth structure of the channel, i.e., the polymer distribution along the channel depth, whereas off-specular signals resolved the lateral brush structure within the channels.

Notably, in neutron reflectometry measurements, the scattering potential of materials is determined by their scattering length density (SLD), the neutron scattering equivalent of the optical refractive index. Accordingly, fits to the specular NR data enabled nanometer-level characterization of the SLD along the channel depth, providing detailed characterization of the channel thickness and the fraction of polymer brush decorating the channel walls. In fitting reflectometry data, the sample is typically modeled as contrasting layers and the thickness and SLD of each layer are used as fit parameters. The layer composition is then indirectly inferred from the fitted layer SLD. However, investigating brush conformations within the channel requires access to the lateral structure. Here, we note that specular signals are determined by the laterally averaged channel SLD (Figure S1), which is insensitive to the brush conformation but rather depends on the total volume fraction of the brush within the channel (see Data Fitting and Analysis in Supporting Information). To address these limitations, we performed complementary off-specular measurements, as discussed below.

### 2.1 In-Depth Channel Geometry

The channel parameters obtained from specular fits under different solution conditions are shown in Table 1. Our fits yielded an average total channel depth of ≈266 nm, in agreement with atomic force microscopy (AFM) measurements (see Figure S2). The total channel depth includes the chromium layer deposited on the top and bottom surfaces of the channels to confine brush growth to just the channel walls as well as the wall depth on which the brushes were grown. The slight variation in the total channel depth among different samples can be primarily attributed to the top layer’s roughness influenced by the brush conformation in the vicinity of the surface, causing minor changes in the overall apparent thickness. A schematic of the different layers used in the data fits is shown in Figure S1.

**Table 1:** In-Depth Fit Parameters Obtained from the Analysis of Specular Reflectivity Data^a^

sample	channel depth [nm]	*ρ*_b_^av^ [10^–6^ nm^–2^]	*d*_b_ [nm]	*f*_p_ [%]	*ρ*_sol_ [10^–6^ nm^–2^]
PH 10	262.2 ± 0.8	3.3	221.9 ± 0.2	29	6.1
PH 4	265.8 ± 0.1	3.3	219.7 ± 0.1	29	6.0
PH 4, 10 mM	263 ± 1.3	3.2	216.8 ± 0.8	30	5.9
PH 4, 100 mM	269 ± 1.4	3.2	221 ± 1.3	30	5.9
PH 4, 1 M	270.4 ± 0.3	3.2	221.7 ± 0.2	29	5.9

a Here, *ρ*_b_^av^ and *d*_b_, respectively, indicate the average SLD and the depth of the brush-decorated layer of the channel. *f*_p_ is the volume fraction of the polymer within the channel, and *ρ*_sol_ is the SLD of the deuterated buffer solution at each condition.

Another outcome of the specular analysis is the ability to accurately determine the solution SLD at each pH and salt condition, which is needed to determine the brush SLD and the corresponding changes in the brush conformations from off-specular analysis. Note that small variations in the solution SLD are expected during pH adjustment or upon the introduction of salt. In this study, we determined the solution SLD from the critical edge in the specular signals at which a sharp drop from unity is observed in the specular intensity. The wavevector transfer, *Q*_c_, describing the critical edge is given by the SLD difference between the incident medium (silicon) and the reflecting medium (deuterated solution) such that . Knowing the SLD of silicon (*ρ*_Si_ = 2.07 × 10^–4^ nm^–2^), we were able to accurately calculate the solution SLD under each of the conditions studied here. These values were subsequently used in calculating the total volume fraction of the polymer, *f*_p_, within the channel, found to be ≈30%.

### 2.2 Brush Conformations

**Figure 2:**
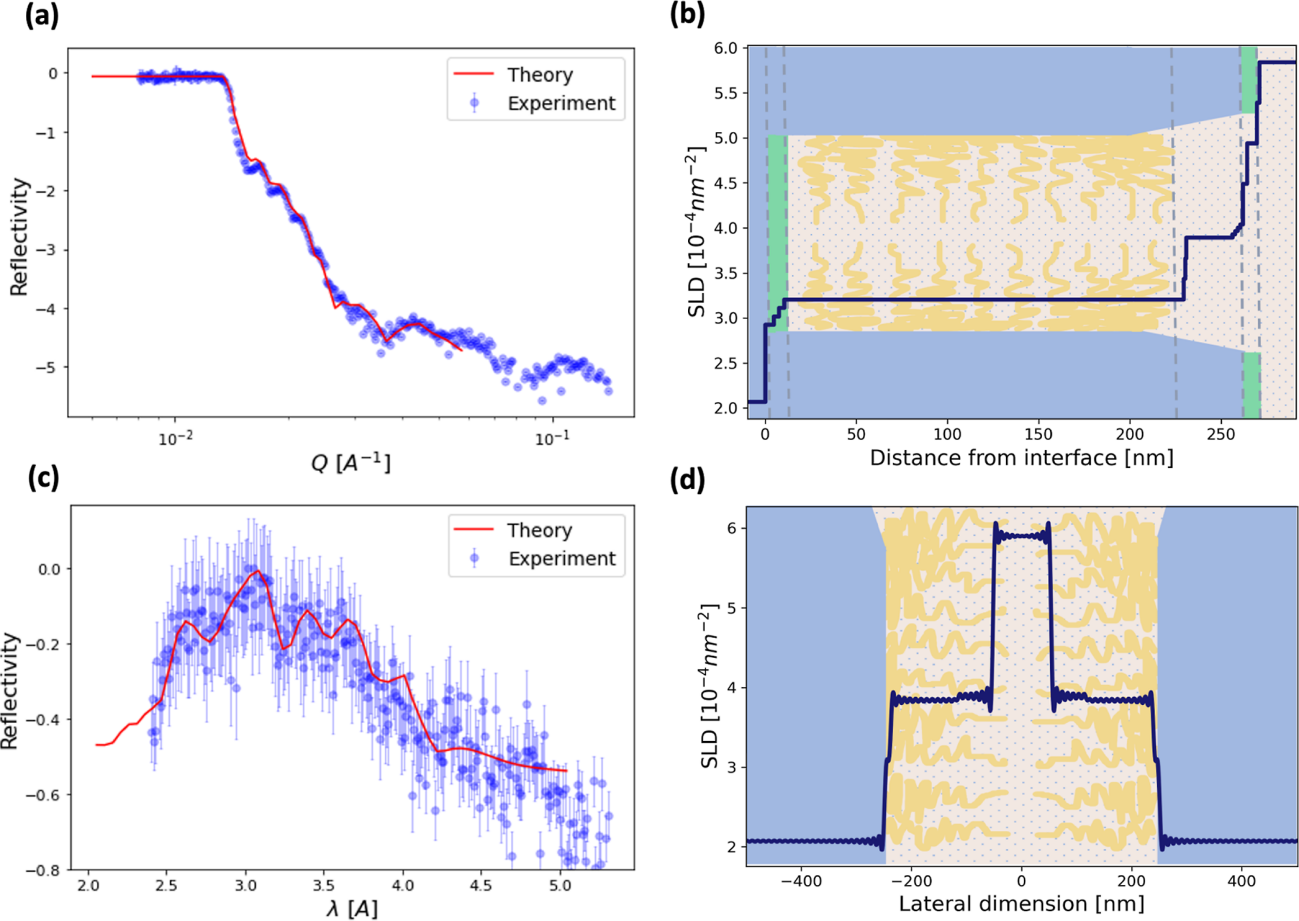
Simultaneous fits of the specular and off-specular signals from the PDMAEMA-functionalized channels at pH 4 and 1 M salt concentration. Fits to the (a) specular and (c) off-specular signals yielded the (b) average neutron SLD profile as a function of channel depth and the (d) lateral SLD profile of the channel layer containing the polymer brushes. In panel (d), the highest SLD value at the center of the channel represents the deuterated solution or the gate opening between the polymer brushes on the opposite sidewalls of the channel. The two adjacent plateaus represent regions with different volume fractions of the polymer brush, indicating a dense region near the sidewalls and a less compact region farther from the walls. The error bars represent ±SD.

Brush conformations were obtained from simultaneous fits to the specular and off-specular signals, enabling us to retrieve the 3-dimensional structure of the polymer brush layer coating the channel sidewalls (Figure 2). A key outcome of these fits is the lateral SLD profile of the channel, as shown in Figure 2d, which was subsequently used to determine the polymer volume fraction away from the channel walls.

**Figure 3:**
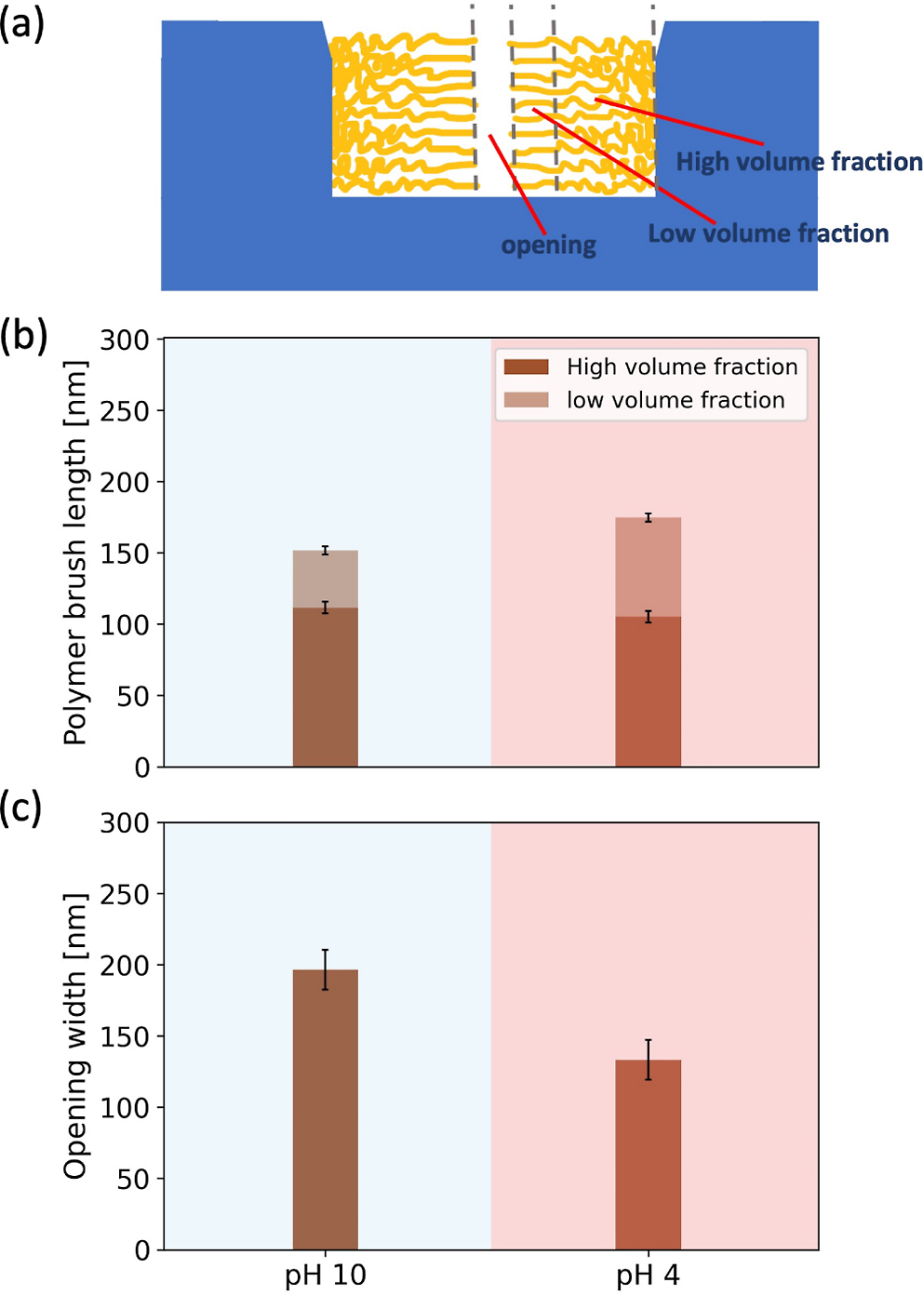
Brush response to variation in solution pH with no added salt. (a) Schematic of a nanofluidic channel showing the high and low volume fraction regions of the polymer brush and the total thickness of each layer. (b) Influence of the pH level on the length (or thickness) of the high and low volume fraction regions. (c) Effect of the pH level on the nanofluidic channel opening. The error bars represent ±SD.

**Figure 4:**
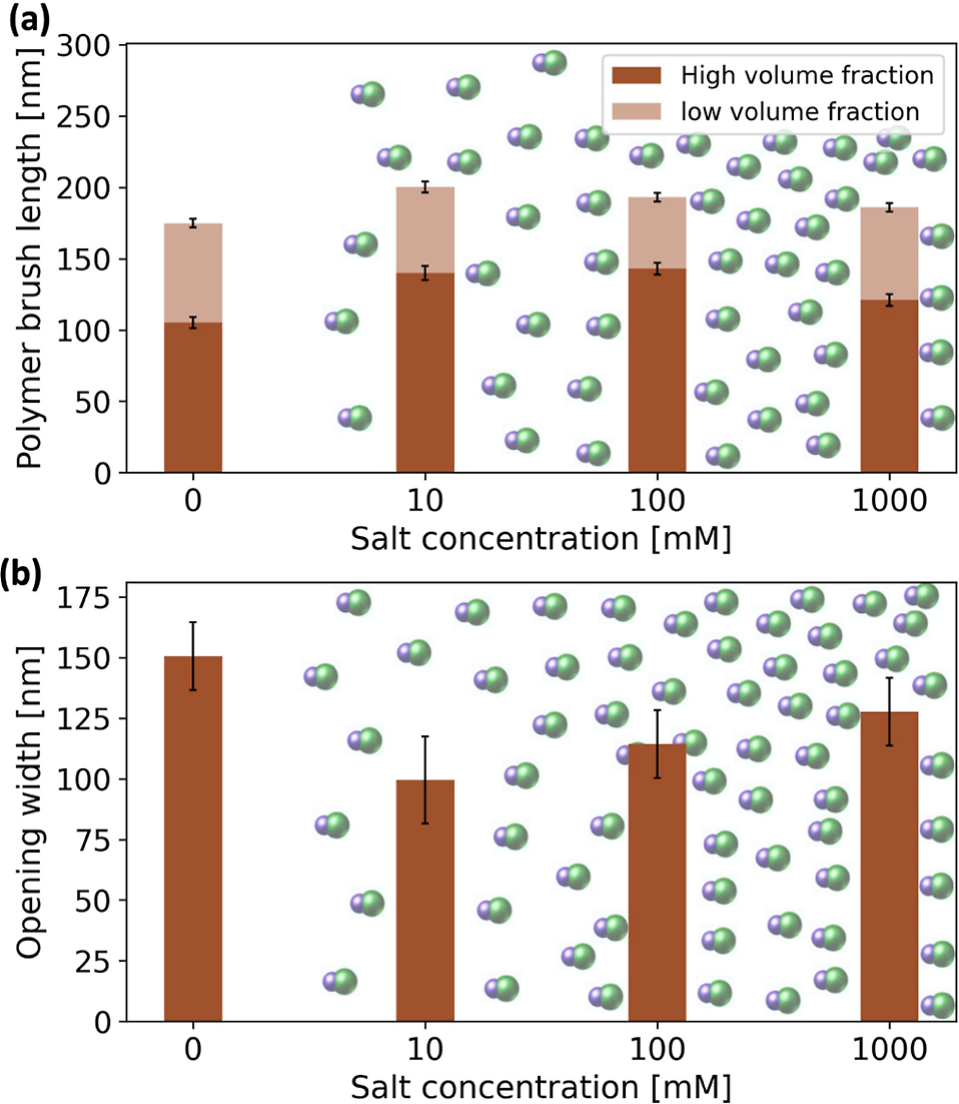
Brush response to variation in salt concentration at pH 4. (a) Influence of salt concentration on brush conformations. (b) Effect of salt concentration on the nanofluidic channel opening width. At low salt concentrations (osmotic regime), the brush increases in thickness by increasing ionic strength. However, the brush decreases in thickness at high salt concentrations (salted brush regime). The error bars represent ±SD.

Fits to the specular and off-specular data sets obtained at different pH values and salt concentrations are shown in Figures S8 and S9. Our data fits show that the brush layer is best described by two regions, a dense region near the channel walls (i.e., the grafting interface) and a relatively less compact region farther from the interface. More importantly, the thickness and SLD of these two regions varied with the solution pH and salt concentration, as discussed below and as shown in Figures 3 and 4.

To validate the need for a two-region brush model, we briefly discuss the common structural and conformational properties of polymer brushes near interfaces. Specifically, in densely grafted brushes, polymer chains tend to undergo excluded volume effects and experience strongly stretched conformations compared to free polymer chains.^39^ For neutral brushes, the thickness of the brush as a function of grafting density scales as *L* ∝ *N*σ^1/3^ as derived by Alexander^40^ and de Gennes.^41^ When polymer chains contain charged monomers, i.e., polyelectrolytes, the electrostatic interactions and subsequent counterion adsorption lead to an increased osmotic pressure of confined counterions within the brush. As a result, the scaling behavior of polyelectrolyte polymer brushes is determined by the contribution of repulsive interchain interactions and the counterion osmotic stretching force. “Strong” polyelectrolyte brushes are generally “quenched”, such that the degree of ionization of the grafted chains remains largely unaltered since the solution conditions hardly change the number of charges on the chain.

On the other hand, for “weak” polyelectrolytes (e.g., PDMAEMA), the degree of ionization can be adjusted by varying the solution conditions, mainly via the solution pH and ionic strength.^42^ In this case, the excluded volume contribution increases with grafting density, while the osmotic stretching force due to counterions is independent of the grafting density. Accordingly, the volume change of a polyelectrolyte brush to the solution conditions is more prominent in low-density brushes.^33^ In such systems, theoretical models predict a Gaussian density profile in the “electrolyte” regime and a parabolic density profile in the “quasineutral” regime.^43^ The former density profile is accounted for by a term responsible for the counterion entropy, while in the latter case, the swelling is contributed by the repulsive volume interchain interactions. Such behavior has been observed in previous empirical studies,^26,27,33^ indicating nearly constant polymer volume fraction for thin polymer brush layers but variable distribution within thicker brushes, with a higher polymer density close to the grafted surface and lower density away from the surface. This behavior can be partially attributed to chain-length polydispersity or the increased conformational space for polymer chains in the outer region of the brush layer.

In addition, the formation of hydrophobic domains potentially due to globule conformations can result in relatively denser regions close to the surface.^37^ While for free chains such globular conformations can be observed anywhere on the backbone, for grafted brushes they form favorably close to the grafted surface to allow a gain in interfacial energy.^44^ Therefore, a thin layer with high monomer volume fraction was included close to the channels’ sidewalls, where the brushes were initiated to account for the attractive interaction between initiators and PDMAEMA segments.^33,38^ The two other pronounced regions of the brush with different SLDs (Figure 2d) are directly related to the monomer volume fraction within each region. Considering ρ_sol_ reported in Table 1 and ρ_polymer_ = 0.8 × 10^–4^ nm^–2^,^33^ our measurements indicated a high volume fraction region near the sidewalls, and a less compact region far from the grafting surface. Beside the brush thickness, the computed SLD profile was used to determine the gap or opening width of the channel under different solution conditions. Therefore, our approach provides a unique noninvasive method for characterizing the effective gate opening in a tunable nanofluidic channel in the unperturbed state of the polymer brush and under different solution conditions.

### 2.3 Effect of pH on Gate Opening

We performed pH-variation experiments, under basic (pH 10) and acidic (pH 4) solution conditions, i.e., above and below the free-polymer p*K*_a_ (≈6), respectively, to explore the channel gating response to conformational changes of the PDMAEMA brushes. Here, it is important to point out that the brush p*K*_a_ can differ from that of a dilute solution of the free polymer due to the grafting density or salt content.^34^ However, the pH values in this study are well above and below the free-polymer p*K*_a_ to ensure different conformational states of the polymer brush. The PDMAEMA brush contains dimethylamino groups which determine the charge of the grafted chains by the level of association or dissociation of protons at various pH values.^45−47^ At acidic conditions (pH 4), the brush assumes a swollen conformation due to the protonation of the amino groups, leading to a high osmotic pressure of the counterions entrapped in the brush domain.^26^ However, under basic conditions (pH 10), the amino groups become deprotonated and result in a collapsed brush configuration.

Figure 3 shows the thicknesses of the dense and less compact regions of the brush and the resultant gate opening (see Table S2 for the tabulated parameters). In these measurements, the total brush thickness at pH 10 was found to be 152 ± 7 nm, with a dense brush region of ≈112 nm and a low-density region of ≈40 nm. In comparison, at pH 4, the total brush thickness was found to be 175 ± 6 nm, indicating brush swelling due to the osmotic effect of counterions. The dense and dilute brush regions were found to be ≈105 and ≈70 nm thick, respectively, showing expansion of both brush regions. Similar swelling behavior was observed in previous studies of PDMAEMA brushes on flat substrates using specular neutron reflectivity.^27,33,37^ However, these studies report higher swelling ratios compared to those obtained here, primarily due to the much lower brush grafting density relative to our study. This has important implications for nanofluidic applications. For example, the grafting density can be tuned to modify how and to what extent changes in pH (and ionic strength) influence the channel gating behavior. Nonetheless, considering the nontrivial change in the brush thickness from the opposite sidewalls between pH 10 and pH 4, we observed a significant reduction in the gate opening from ≈196 to ≈150 nm, i.e., the equivalent of ≈46 nm narrowing in the effective channel opening. This indicates a switching of the channel geometry from an open state to a semiclosed state. Importantly, the dependence of the gate opening on the pH level can be further modified by controlling the length of the grafted chains or the grafting density.

Our results are also consistent with earlier in situ measurements of PDMAEMA brushes showing sharp and reversible switching between swollen and collapsed states on flat substrates.^26^ While our measurements did not allow for repeated pH cycles (due to limited beamtime), experiments on the kinetic behavior of similarly weak polybase polymer brushes, poly(2-(diethylamino)ethyl methacrylate)(PDEA), indicate highly reproducible changes between swollen and collapsed states over several pH cycles.^48^ Such robust dynamic switching behavior of weak polybasic brushes enables active control of the gate opening in nanofluidic channels by regulating the solution’s pH condition with a precision of a few tens of nanometers.

### 2.4 Effect of Ionic Strength on Gate Opening

In addition to pH variation, we also investigated the effect of variations in ionic strength on the brush conformations and resultant channel geometry. For cationic polyelectrolytes, like PDMAEMA, the impact of salt concentration is particularly significant under acidic conditions. This heightened charge density leads to stronger electrostatic interactions, affecting the osmotic pressure of the counterions within the brush.^45,49−51^ To further understand this phenomenon, we studied the effect of ionic strength on the brush structure at pH 4, i.e., in the extended brush regime, for salt concentrations of 10, 100, and 1000 mM.

The ionic strength influences the electrostatic interactions in the brush region by regulating the electrostatic screening length, and also by adjusting the number of charged monomer groups in the brush layer.^52^ The response of the brush thickness to the ionic strength is highly dependent on the brush grafting density and the charge fraction of the chain.^53^ Theoretical studies by Zhulina et al., based on mean field theory, predict that for a relatively low grafting density in the polyelectrolyte regime (where electrostatic interactions are dominant compared to volume interchain interactions), the brush thickness does not scale monotonically with the salt concentration (*c*_s_).^54^ Mainly, in the “osmotic” regime, when the salt concentration in bulk solution is smaller than that of the counterion inside the brush, the brush thickness scales as *L* ∝ *c*_s_^1/3^.

In the opposite limit of the “salted” brush regime, salt ion charges dominate immobilized charges inside the brush and brush thickness scales as *L* ∝ *c*_s_^–1/3^. Similar chain behavior was reported by Fleer based on a self-consistent field theory (SCFT) model^55^ in the case where the excluded volume effect is weak, i.e., when *w*^1/2^σ*a*^2^*m* ≪ 1, where *w* is the third virial coefficient, σ is the grafting density, *a* is the size of polymer segments, and *m* is the elementary charge on each chain.^43^ Witte et al. applied continuum numerical SCFT to demonstrate that such classic scaling profile applies at very low chain density, when the effective segment volume is negligible.^49^ Within theoretical limits, previous theoretical^50,53,54^ and experimental studies^42,48,56^ have reported a qualitatively similar trend for dense brushes, specifically that the thickness of the polyelectrolyte brushes increases with salt concentration in the osmotic regime and decreases in the salted brush regime.

The results from our study, summarized in Figure 4a, show that the response of the polymer brush to the variation in the salt concentration follows a similar behavior. An increase in the total thickness of the brushes was observed by adding 10 mM salt compared to the salt-free solution, resulting in a swelling of the brush from 175 ± 6 to 200 ± 8 nm. In this regime, by adding salt, the anions take the place of hydroxide counterions within the brush and, as a result, increase the degree of dissociation which in turn increases the osmotic pressure inside the brush. On the other hand, at high salt concentrations (100–1000 mM), we found that the brush exhibited a slight decrease in the total thickness from 193 ± 7 to 186 ± 6 nm. This indicates a more moderate change compared to the case of low salt concentration (0–10 mM). At such relatively high ionic strengths, the bulk electrolyte concentration surpasses the concentration of counterions inside the brush, and any addition of salt increases the screening of the electrostatic interaction between monomer groups which can decrease the brush thickness. Therefore, we observed a smaller thickness of the brush in the highly salted case (1000 mM) compared to the case of low salt concentration, in agreement with results from previous experimental studies.^48,56−58^

Our findings also indicate that even when the solution is highly salted, the average brush length remains higher than in the salt-free solution. While these findings qualitatively agree with the theoretical predictions and experimental observations discussed earlier, they do not precisely obey the classic scaling laws indicating a sharp increase in brush thickness at low salt concentration (power of 1/3) and a sharp decrease in the high salt limit (power of −1/3) (see Figure S10). Such observed difference can largely be explained by the high grafting density (σ > 0.5 nm^–2^) in our experiment, which results in significant interchain interaction effects resulting in deviations from the “electrolyte” regime predicted by Zhulina et al.^54^ This quantitative discrepancy between empirical measurements and classical predictions on weak polyelectrolytes^48^ may be also attributed to other factors, such as nontrivial three-body interactions at high grafting density, chain polydispersity, or polymer backbone hydrophobicity which were not considered in previous theoretical calculations.^59^

Nonetheless, the resultant changes in brush conformation led to changes in the effective channel opening, indicating that regulation of the ionic strength can be used as an additional control for the gate opening (see Figure 4b). Specifically, the increase in salt concentration to 10 mM caused a reduction in the gate opening from ≈150 to ≈100 nm, i.e., a more closed state. However, in the high salt concentration, the opening width increased from ≈114 nm at 100 mM to ≈128 nm at 1000 mM, due to the weaker dependence of the brush thickness at high salt concentrations. Yet, adding a high amount of salt kept the gate in a semiclosed state compared to the nonsalted situation.

Interestingly, previous studies have shown that polyelectrolyte brush response to the ionic strength at pH level below p*K*_a_ is more symmetric across the swollen and the collapsed state, with a considerably faster collapse conversion rate compared to the pH-induced variation.^48^ This is not completely unexpected, since the ionic strength controls the pH response of weak polyelectrolytes by regulating the screening of electrostatic repulsion. As the salt concentration increases, a higher pH is required to trigger brush collapse. The time needed for the brush to collapse also increases in the presence of the excess electrolyte due to a slower diffusion rate of ions from within the brush to the bulk. Overall, such observations indicate that changes in the ionic strength can still modify chain conformation, directly and indirectly by influencing the pH response. This points to the potential for novel mechanisms of gate control by utilizing a combination of pH and ionic strength to regulate the thickness and rate of change in the brush.

## 3 Materials and Methods

### 3.1 Sample Preparation

**Figure 5:**
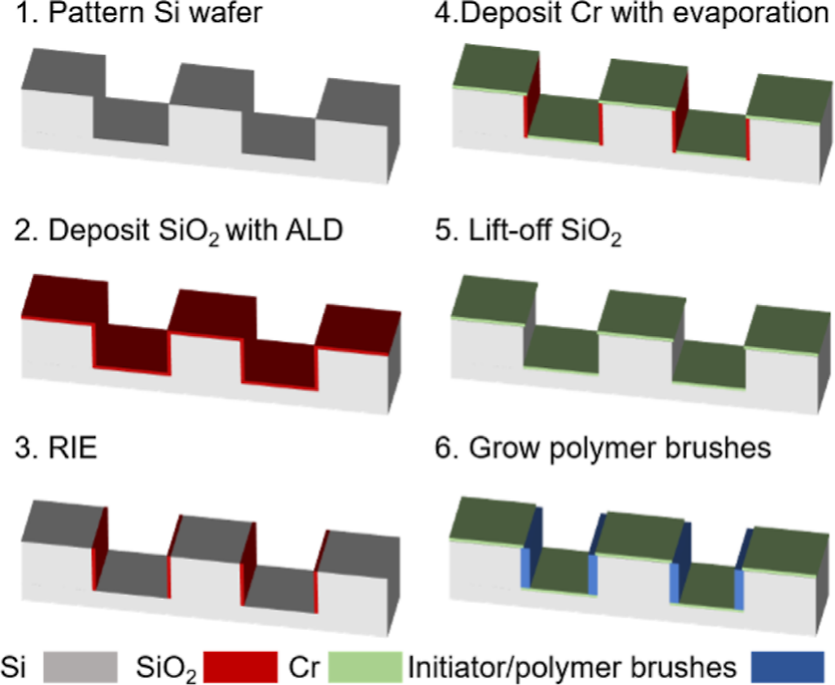
Schematic of the different steps used in the fabrication of the channels and their functionalization with polymer brushes.

A schematic description of the different processes used in sample preparation is shown in Figure 5.

#### 3.1.1 Materials

Double-sided polished silicon wafers (100) with a thickness of 0.7 mm were purchased from El-Cat Inc. Allylamine, anhydrous toluene, α-bromo-isobutyryl bromide (BIBB), chlorodimethylsilane, copper bromide(CuBr), dimethyl sulfoxide (DMSO), inhibitor remover (for removing hydroquinone and monomethyl ether hydroquinone), magnesium sulfate, (2-dimethylamino)ethyl methacrylate (DMAEMA), Pt on activated carbon (10 wt %), triethylamine, bipyridine, 2,2′-bipyridine, pyridine, sodium chloride, and sodium phosphate monobasic monohydrate were purchased from Sigma-Aldrich and used without further purification unless stated otherwise. All the other solvents and chemicals were purchased from Fisher Scientific.

#### 3.1.2 Initiator Synthesis and Immobilization

First, 2-bromo-2-methyl-*N*-allylpropanamide and chlorodimethylsilane were synthesized following published procedures and used as a subsequent reactant.^60^ Hydrosilylation was then carried out using another literature procedure to obtain the monofunctional ATRP initiator, 2-bromo-2-methyl-*N*-{3-[chloro(dimethyl)silyl]-propyl} propanamide.^60,61^ Substrates were cleaned by repetitive dichloromethane (DCM) and DI water rinsing and then dried with nitrogen. The substrates were subsequently oxidized using a Harrick Plasma Cleaner for 10 min. Moisture was further removed by 10 min of 110 °C oven baking. Using a glovebox, the substrates were immersed overnight at room temperature in a toluene solution of the initiator (10 mM) and pyridine (0.5 mM). The substrates were then removed from the solution and washed with DI water and DCM sequentially, followed by a final rinse with dichloromethane and drying under nitrogen gas.

#### 3.1.3 Synthesis of the PDMAEMA Brushes

PDMAEMA monomer solution was first passed through an inhibitor remover column to remove monomethyl ether hydroquinone. The monomer (10 mL, 68.2 mmol) was then mixed with DMSO (10 mL, 116 mmol) and injected in a closed Schlenk flask with argon bubbling for more than 30 min. CuBr (93 mg, 0.65 mmol) and 2,2′-bipyridine (207 mg, 1.33 mmol) were stirred in another 25 mL Schlenk flask using a magnetic stir bar. Another Schlenk flask was equipped with initiator deposited substrates. The air in both flasks was evacuated and replaced with argon three times. The solvent mixture was then cannulated into a flask containing the ligand and copper salts. The reaction mixture was stirred at room temperature for more than 30 min to ensure the dissolution of the monomer and the copper-ligand complex in the solvent. This solution was then transferred to the Schlenk flask containing the initiator-deposited substrates. Polymerization was carried out for 2 h at 60 °C. After polymerization, the substrates were removed from the flask and washed with ethanol and dried under a stream of nitrogen.

#### 3.1.4 Fabrication of the Periodic-Channel Substrates

Silicon wafers were cleaned with hot piranha solution and washed with water before nitrogen blow drying. Bottom antireflective coating (BARC), DUV 42-P and UV210 photoresist were spin-coated and subjected to corresponding postapplication bake. Photolithography was conducted with an ASML 300C DUV stepper to form a pattern on the UV210 photoresist. Reactive ion etching (RIE) was then conducted on an Unaxis SLR 770 to transfer pattern into the silicon. Residual photolithographic materials were then removed by RIE in an Oxford PlasmaLab 80+ RIE System. SiO_2_ was then conformally deposited with Oxford ALD FlexAL at 110 °C, 80 mTorr with the help of plasma to obtain the required sacrificial layer (used trisdimethylaminosilane, rate = 0.827 nm/cycle). The SiO_2_ layers on the top and bottom of the trenches were then removed by RIE with CHF_3_/O_2_ gas mixture in Oxford PlasmaLab 80+ RIE System, and the substrates were then cleaned with RIE in the same system. Deposition of the Cr was conducted on a CVC SC4500 E-gun Evaporation system with a deposition rate ∼0.5 nm/s. The temperature of the substrate was continuously monitored, and it was always below 25 °C during the process at 3 × 10^–6^ Torr. Lift-off was conducted by immersing the substrates in HF or buffered oxide etching for a given time. The samples were then washed with copious amounts of water to remove the residual etchant. After drying in 110 °C oven for 10 min, substrates were then subject to plasma clean in Harrick Plasma Cleaner for 7 min before put into the solution for initiator deposition. The following cleaning and polymer brush synthesis procedures were the same as previously described.^68^ Additional etching condition settings could be found in Supporting Information.

### 3.2 Neutron Reflectometry Measurements

Neutron reflectometry experiments were carried out on the liquid reflectometer (BL-4B) at the Spallation Neutron Source (SNS) at the Oak Ridge National Laboratory. The measurements were performed in two scattering geometries: specular and off-specular. Specular data were collected using standard beam apertures and slit sizes to obtain a specular reflectometry signal over a *Q*-range of 2 × 10^–3^ to 2 × 10^–2^ nm^–1^. In off-specular measurements, the detector was offset by 4°, and the specular beam was masked (as shown in Figure 1) to enable long acquisition times of the Bragg rods, which are typically several orders of magnitude weaker than the specularly reflected beam. Measurements were conducted using a custom-made fluid cell comprising a thick silicon puck with a sample compartment and a quartz window with two microfluidic ports for solvent exchange. Details of the fluid cell design are described in previous work.^29^ The microfluidic ports are designed with lever fittings that enable solution injection or disposal using syringe connections. To achieve optimal neutron contrast, reflectometry measurements were performed using D_2_O buffers with the target pH and salt concentration. All studies were conducted on the same substrate with brush-functionalized channels, and changes in the solution conditions were achieved by copious flushing of the cell with the target solution, without disassembling the cell. More specifically, for each target pH or salt concentration, the cell was flushed with 5 mL of the target solution, i.e., the equivalent of 10 times the cell volume (0.5 mL), to ensure solution exchange. Measurements with salted solutions were conducted with increasing salt concentrations. After each flushing, the sample was left to equilibrate for at least 30 min before starting the specular runs, which typically require 1.5 h of measurement time. The off-specular measurements were performed right afterward, thus providing 2 h of brush equilibration time prior to off-specular data acquisition.

### 3.3 Data Fitting Procedures

#### 3.3.1 Specular Data Analysis

Specular neutron reflectivity data were initially analyzed using the Motofit package^62^ to determine the thickness, roughness, and average SLD of the different layers within the sample. These fits were later used to build the initial SLD profile in the dynamical theory (DT) model utilized in simultaneous fitting of the specular and off-specular signals (see the Dynamical Theory Model section in Supporting Information). A comparison of the specular reflectometry calculations by Motofit and Dynamical Theory is shown in Figure S5. Since the measurements were done such that the neutron beam was incident from the silicon substrate, the data was modeled with a front layer representing the silicon substrate (SLD = 2.07 × 10^–4^ nm^–2^) and a back layer representing the deuterated solution. To model the channel structure, three or four layers were considered as shown in Figures S1 and 2b. A comparison of the two models is shown in Figure S3. In the case of four layers, the fourth layer represented a thin layer consisting of a chromium layer on the sample surface (to prevent brush growth from the surface) and an intermediate solvent in the channel region (see Figure S1 for more details). The data fits showed better agreement with the data in the four-layer model, as evident from the fit residuals (χ^2^ = 0.0198 for the 3-layer model vs χ^2^ = 0.0187 for the 4-layer model). The comparison is shown for the case of pH 10 in Figure S3. To account for interfacial roughness,^69^ we introduced a thin-slicing formalism that was necessary for capturing the drop in intensity caused by roughness (see Figure S6).

#### 3.3.2 Off-Specular Data Analysis

The lateral characteristics of the channels and the polymer brush, e.g., channel width and brush thickness, cannot be resolved from laterally averaged specular reflectometry data but can be obtained from the off-specular signals. Earlier studies have shown that the intensity variation of the off-specular signals as a function of neutron wavelength embeds unique information of the lateral structures and SLD profile of the sample.^63^ These signals are analyzed using a dynamical theory (DT) model that has been validated in previous experiments^35,64,65^ on periodic channel structures, like those used in the current work. The details of the data reduction are described in Data Reduction for Off-Specular Signals of Supporting Information and are demonstrated graphically in Figure S7. The data fitting was performed by using an optimization protocol described below.

#### 3.3.3 Optimization Protocol

Simultaneous fits of the DT model to the specular and off-specular neutron data were performed by using an optimization protocol that fits key parameters of the sample profile, including brush thicknesses and SLDs. When possible, starting values of certain parameters (e.g., channel depth and width) were set to their experimentally observed values by other methods, including AFM measurements. Other parameters, such as the interfacial roughness, were input from the analysis of the specular signals by Motofit. In addition, we considered two regions of the polymer brushes away from the channel sidewalls. This was inspired by earlier studies showing different conformational states of polymer brushes away from interfaces.^33^ These two regions were assigned independent fit parameters for their thickness and SLD. Subsequently, the fit optimization was done using the Covariance matrix adaptation evolution strategy (CMA-ES) package in python.^66^ We defined a weighted total χ^2^ from specular and off-specular data fits as a fitness function (η) to show the goodness of the fit to the DT model. For parameters estimated from other measurements or from specular fits, the initial value of the parameter and its range were defined and were used to set an upper and a lower bound on the fit values. The CMA-ES package generates a set of *n* random parameters within the specified range as training sets of variables. Based on the fit residuals of each parameter set, the CMA-ES algorithm predicts a new set of parameters to be calculated for a new value of η. This procedure is iteratively repeated until η converges.

## 4 Conclusions

This study presents a practical design for nanofluidic channels featuring controllable gating properties, through the functionalization of submicrometer channel walls by PDMAEMA chains. The top and bottom surfaces of the channels were masked by chromium, restricting brush growth to just the sidewalls. This design permits channel gating in a controllable manner by utilizing the conformational change of the polymer brush in response to solution pH and ionic strength. Specifically, collapsed brushes result in an open gate state, while swollen brushes lead to a semiclosed gate structure. To investigate the brush conformation state, we performed specular and off-spectra neutron reflectivity measurements. By fitting the reflected signals using dynamical scattering theory, we obtained a 3D view of the channel under varying pH and salt concentrations. The results indicate that decreasing the pH level below the polyelectrolyte p*K*_a_ leads to an expanded brush conformation. Furthermore, the addition of salt initially causes brushes to expand further in the “osmotic” regime, followed by a slow decrease in thickness in the highly “salted” brush regime. The results show a deviation from the classic scaling laws of brush thickness with salt concentration, which can be attributed to the high grafting density of the polymer brush in this current study. While high grafting density is typically seen as a favorable feature for nanofluidic applications, we conjecture that it can also result in a weaker gating response to pH and salt concentration. In conclusion, with the increasing need for developing active valving components with regulated nanofluidic flow,^67^ this study offers a noninvasive means for exploring the gating response of polymer functionalized nanochannels to solution conditions with high sensitivity and precision.

## Supporting Information Available

Detailed experimental procedures (substrate etching, brush growth and characterization), data fitting and analysis methods (model validation and theoretical description), image processing and data reduction protocols, additional plots of data and data fits, and tabulated fit parameters (PDF)
