# Supplementary material for: Synthesis and Characterization of Stimuli-Responsive Polymer Brushes in Nanofluidic Channels
Source: ACS Appl Mater Interfaces. 2023 Nov 16;15(47):54942–51. doi: 10.1021/acsami.3c12744 (PMC10695172; doi:10.1021/acsami.3c12744)
Supplement: Supplementary file 1 — am3c12744_si_001.pdf [file am3c12744_si_001.pdf]

Supporting Information

Synthesis and Characterization of Stimuli-Responsive Polymer Brushes in Nanofluidic Channels

Hadi Rahmaninejad,[†,‡] Andrew J. Parnell,[¶] Wei-Liang Chen,[§] Nilay Duzen,[‖] Thomas Sexton,[¶] Gary Dunderdale,[⊥] John F. Ankner,[#] Wim Bras,[@] Christopher K. Ober,[*,‖] Anthony J. Ryan,[*,△] and Rana Ashkar[*,∇,‡,††]

†*Department of Physics, Virginia Tech, Blacksburg, VA 24061, USA*

‡*Center for Soft Matter and Biological Physics, Virginia Tech, Blacksburg,VA 24061, USA*

¶*Department of Physics, The University of Sheffield, Sheffield S3 7RH, United Kingdom*

§*Department of Material Science and Engineering, University of Pennsylvania, Philadelphia, PA 19104, USA*

‖*Department of Material Science and Engineering, Cornell University, NY 14850, USA*

⊥*Department of Chemical and Biological Engineering, The University of Sheffield, Sheffield S1 3JD, United Kingdom*

#*Second Target Station, Oak Ridge National Laboratory, Oak Ridge, TN 37830, USA*

@*Chemical Sciences Division, Oak Ridge National Laboratory, Oak Ridge, TN 37830, USA*

△*Department of Chemistry, The University of Sheffield, Sheffield S3 7HF, United Kingdom*

∇*Department of Physics, Virginia Tech, Blacksburg, VA, USA*

††*Macromolecular Innovation Institute, Virginia Tech, Blacksburg, VA 24061, USA*

E-mail: cko3@cornell.edu; a.ryan@sheffield.ac.uk; ashkar@vt.edu

S.1 Materials and Methods

S.1.1 Experimental Details

Substrate etching. Oxford PlasmaLab 80+: For etch of SiO2: CHF3= 50 sccm, O2=2 sccm, 240 W, 40 mTorr. For cleaning: O2=50 sccm, 150 W, 60 mTorr Unaxis SLR 770: Plasma lighting: C4F8=2 sccm, SF6=2 sccm, Ar=40 sccm, O2=10 sccm, ICP=700 W, RIE power=35 W, 8 mTorr, 5 sec. Etch: C4F8=56 sccm, SF6=24 sccm, Ar=40 sccm, O2=10 sccm, ICP=700 W, RIE power=12 W, 8 mTorr.

Characterization of the PDMAEMA brushes grown on flat Si wafers. Thickness of the PDMAEMA brushes grown on flat Si wafer were measured with Woollam VASE spectroscopic ellipsometer with a Cauchy model. To measure the brush molecular weight, PDMAEMA brushes grown on flat Si wafers were etched by THF diluted HF (25 %). After 8 h of immersion the wafers were removed from the HF bath which was then left in a fume hood to dry by evaporation. Free PDMAEMA was collected with ethanol and re-dissolved in DMF after vacuum drying. The collected PDMAEMA was then subjected to DMF-solvent GPC analysis in water GPC with Waters 410 differential refractive index detector.

Polymer brush growth kinetic and grafting density. By controlling the polymerization time, PDMAEMA brushes up to ∼120 nm could be grown on the silicon wafer. As shown in Figure 5 , the polymerization reaction reaches the end after ∼2-3 hours. Further polymerization time cannot increase the thickness of the polymer brushes further. Since it has already been proven in theory that the kinetics of the polymerization are different between bulk and surface-initiated conditions, measurement of the polymer directly cleaved from the brushes is necessary to measure the molecular weight and grafting density.[1] PDMAEMA brushes grown on Si wafer were cleaved by HF immersion and the polymers were successfully collected by ethanol after HF evaporation.

The grafting density is calculated using equation S1 by assuming that the polymer brushes have the same density as in the melt state.

$$h = \frac{M_n \Gamma}{\delta} \tag{S1}$$

In the equation above, Γ is the grafting density, M_n is the molecular weight, and δ is the density of the polymer brush. In this study, M_n = 108 kDa. The molecular weight of the cleaved polymer was determined by gel permeation chromatography based on PS standard calibration. The measurements were conducted in water GPC with Waters 410 differential refractive index detector while DMF was used as eluent. The polymerization time was set to two hours for growing polymer brushes on both Si wafer and periodic-channel substrate. The brush thickness was measured for a planar non-patterned Si wafer using spectroscopic ellipsometry M2000V (J. A. Woollam USA). The measured data was fitted to a Cauchy model resulting in a dry thickness of 94.6 nm. The thickness, grating density, and polydispersity of the brush measured on Si wafer are presented in table S1.

Table. S1: Measured parameters of PDMAEMA brushes.

Polymerization Time	Dry Thickness (nm)	Grafting Density (chains/nm^2)	PDI
2 hours	94.6	0.7	1.08

S.1.2 Data Fitting and Analysis

Specular data analysis using Motofit. Specular analysis was performed using Motofit by considering four sub-layers as shown in Figure S1. Figure S2 shows a specular fit for pH 10 along with the obtained SLD profile normal to the surface. Measurements by AFM of the measured channel containing substrate can be seen in panel (c). An example comparing the fits to the specular signal between the three-layer model and the four-layer model is presented in Figure S3. This figure has been chosen as an illustrative sample for the purpose of demonstration. The difference between the two models is an additional top layer that accounts for the top chromium layer deposited on the grating surface. While the

two models show visually similar fits to the specular data, the 4-layer model yields smaller fit residuals and shows better fits to the offspecular signals.

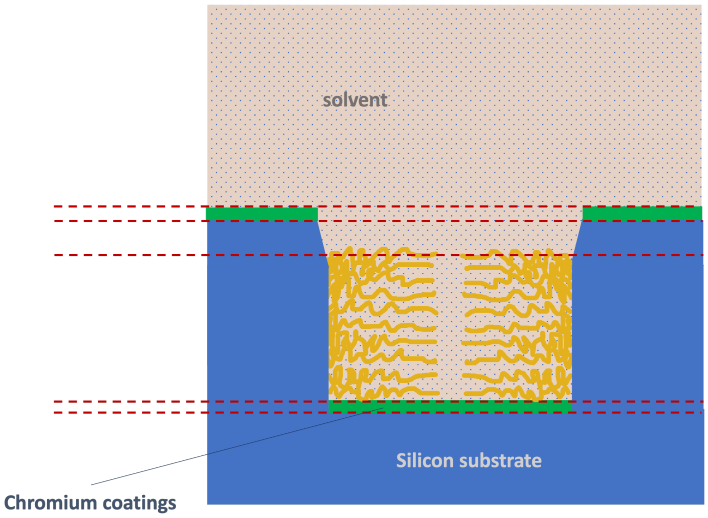

Figure S1: **Schematic of the cross-sectional configuration of the nanofluidic channel functionalized with polyelectrolyte brush**. Figure shows different elements with the four layers considered for specular fittings.

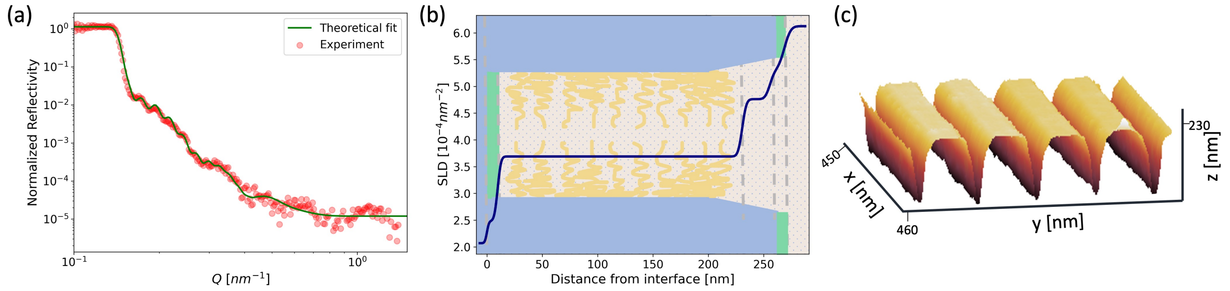

Figure S2: (a) Specular reflection intensity and best fit using a four-layers model in Motofit for pH 10. (b) Average neutron SLD profile obtained as a function of channel depth. (c) AFM image of the wet sample, showing the total thickness and periodicity of the used substrate.

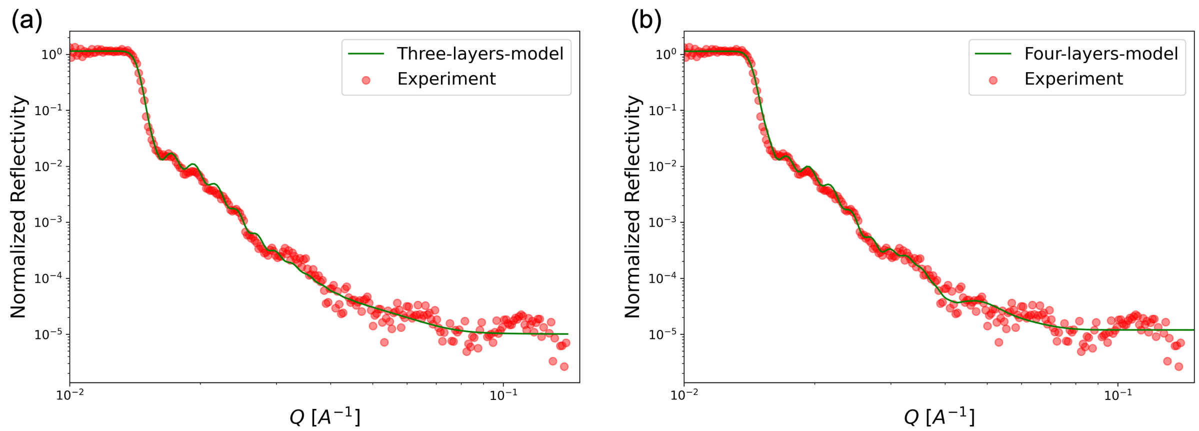

Figure S3: **Comparison of the fits to the specular reflection for three and four layer models.** a) Specular reflection fits for pH 10, using three-layer slicing, and (b) four-layer slicing by the Motofit data fitting package.

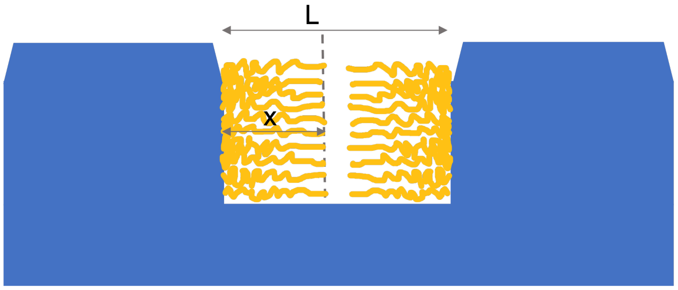

Figure S4: A schematic cross-section of the channel geometry showing polymer brushes grafted onto the sidewalls of a nanochannel, with a brush thickness x and an overall channel width L.

Laterally averaged SLD is independent of brush conformation. The average SLD for the brush region within the channels can be calculated as:

$$\begin{aligned}\rho_{av} &= f * \rho_{Si} + (1-f) * (x(\phi * \rho_{pol} + (1-\phi)\rho_D) + (L-x)\rho_s) \\ &= f * \rho_{Si} + (1-f)(x\phi\rho_p + \rho_s(x(1-\phi) + (L-x))) \\ &= f * \rho_{Si} + (1-f)(c\rho_p + \rho_D(x-c) + (L-x)\rho_D) \\ &= f * \rho_{Si} + (1-f)(c\rho_p + \rho_D(L-c))\end{aligned} \tag{S2}$$

where f is the filling factor of the used substrate, ρ_{Si} is the scattering length density of silicon, and x and ρ_{pol} are the average length and SLD of the polymer brush, respectively.

S.1.3 Dynamical Theory Model

The dynamical theory is employed to predict specular and off-specular reflectivity to retrieve the in-depth and lateral structure of the sample, respectively. The dynamical theory is based on Bloch waves expansion of the neutron wavefunction due to the periodic potential of the sample caused by the channel periodicity[2-4] such that the neutron wavevector in the direction of periodicity is quantized. Components of the wavevectors in the periodic layer and the solution/substrate layers are then computed based on energy conservation. By enforcing the continuity of the wavefunction at every interface, an expression for the reflection and transmission coefficients can be obtained within each layer. Here, we improve on this model by considering interfacial roughness through Parratt slicing of interfaces such that the SLD is gradually changed at the interfaces between the adjacent layers (see discussion below).

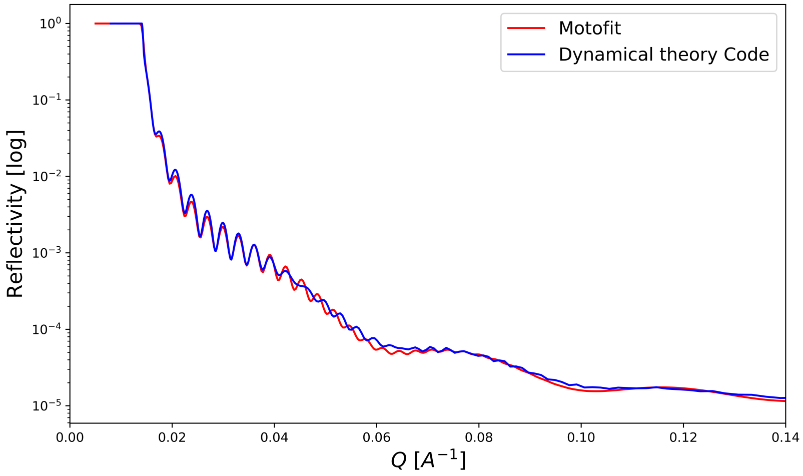

Figure S5: **Comparison between specular reflected intensity using the DT code and the result obtained by Motofit.** The minor discrepancy between these two sets of results arises from the comparatively reduced resolution of predictions by the DT code, compounded by the effects of the smoothing process, particularly noticeable at elevated Q values. This disparity is influenced by the inverse relationship between Q and wavelength.

S.1.4 Validation of DT model

Interfacial roughness. Interfacial roughness was modeled by considering several sub-layers with varying SLD at adjacent interfaces. A larger number of sublayers results in a gradual, more realistic interfacial roughness function. However, increasing the number of sub-layers comes at the expense of computational time. In this study, we modeled interfacial roughness using 5 sublayers. In general, roughness causes an exponential decay in the reflected intensity.[5] We observed similar behavior in the DT model when we considered increasing interfacial roughness (see Figure S6). Including interfacial roughness in this study was necessary for modeling the specular signals and extracting the sample parameters. We find that even with the limited number of sublayers (5) included in our DT model, the DT-calculated specular intensity matches that obtained by Motofit using the same set of sample parameters.

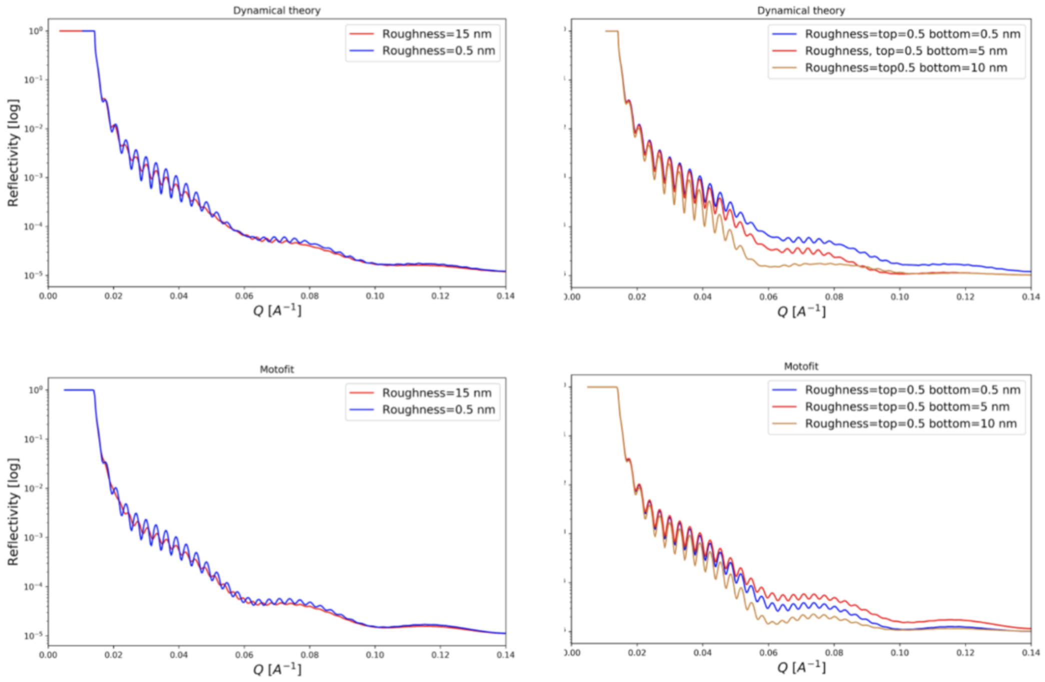

Figure S6: **Validation of theoretical calculations based on DT.** A comparison of the influence of roughness between results obtained from dynamical theory code and Motofit, which validate our theoretical computations.

S.1.5 Data reduction for off-specular signals.

The top left panel in Figure S7 is an example of a detector image obtained from off-specular NR measurements. Data reduction of such signals requires background subtraction which cannot be performed using specular NR protocols available at neutron reflectometers. Instead, we performed background subtraction by choosing a band around the Bragg rod of interest (in this case the 1st Bragg rod) and finding the background intensity as a function of neutron wavelength or time-of-flight. This was done by binning the data in neutron wavelength and modeling the neutron intensity along each bin. Fits of the intensity to a normal distribution (inset in Figure S7c), enabled calculations of the average background intensity along each wavelength bin. The off-specular signal was then obtained by subtracting the background intensity from the total intensity for each wavelength.

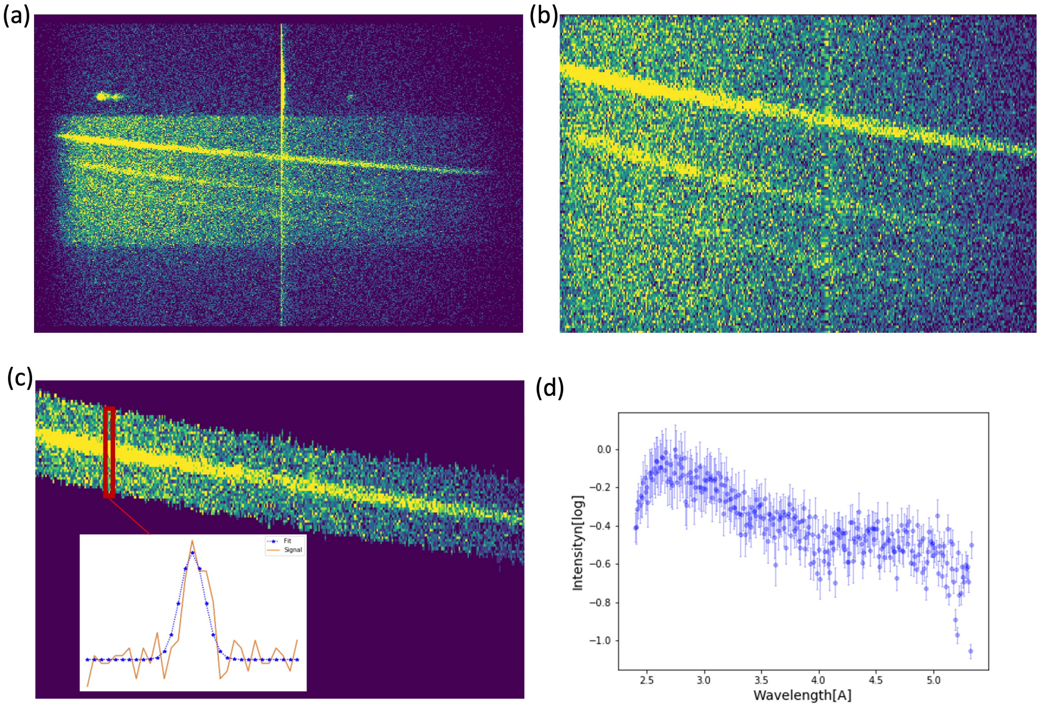

Figure S7: **Data processing for off-specular signals from detector image.** (a) A typical image showing the first few Bragg rods in a time-of-flight experiment. (b) A zoomed-in version of the detector image shown in panel a. c) Depiction of the band chosen around the first Bragg rod. The inset shows a fit of the intensity along the highlighted wavelength bin to a normal distribution function, enabling calculations of the background intensity and subsequent background subtraction. d) 1D representation of the background-subtracted off-specular signal, depicted as intensity vs. wavelength.

S.1.6 Fit Parameters

Table. S2: Lateral brush characteristics obtained at various solution conditions. Here, f represents the filling factor of the substrate, t_{high} and t_{low} are the thicknesses of the high and low volume fraction brush domains away from the channel walls, respectively, with ϕ_{high} and ϕ_{low} representing the polymer volume fractions within each domain.

Sample	f	$t_{high}[nm]$	t_{low}[nm]	ϕ_{high}	ϕ_{low}
PH 10	0.5	112 ± 4	40 ± 3	0.49	0.45
PH 4	0.5	105 ± 4	70 ± 3	0.44	0.37
PH 4, 10mM	0.5	140 ± 5	60 ± 4	0.39	0.35
PH 4, 100mM	0.5	143 ± 4	50 ± 3	0.40	0.35
PH 4, 1M	0.5	121 ± 4	65 ± 3	0.40	0.39

S.1.7 Simultaneous fits to the specular and off-specular signals

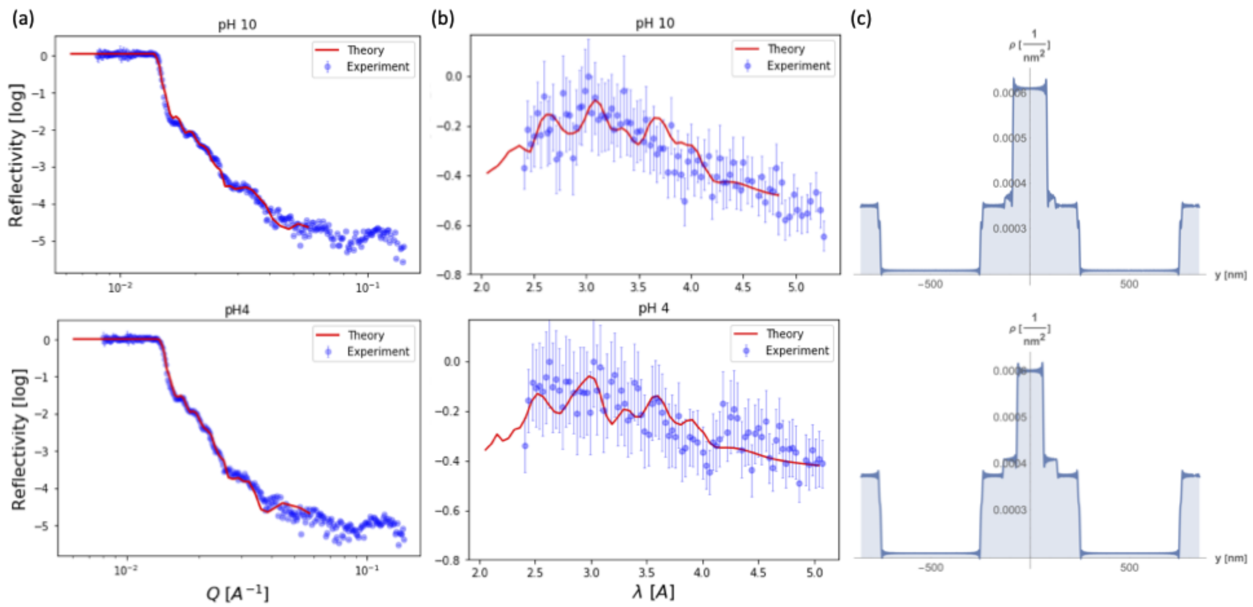

Figure S8: **Simultaneus fits to the specular and off-specular signals for pH4 and pH10** (a) Specular fits (b) off-specular fits, (c) SLD profile of the channel layer containing the polymer brushes. The plot shows a single channel (unit cell) such that $y = 0$ is set to be at the middle of a channel. The middle SLD with the highest value at the center of the channel represents the solvent or the gate between the polymer brushes on the opposite sidewalls of the channel. The two adjacent plateaus represent regions with different volume fractions of the polymer brush, indicating a relatively denser region near the sidewalls and a dilute region away from the sidewall.

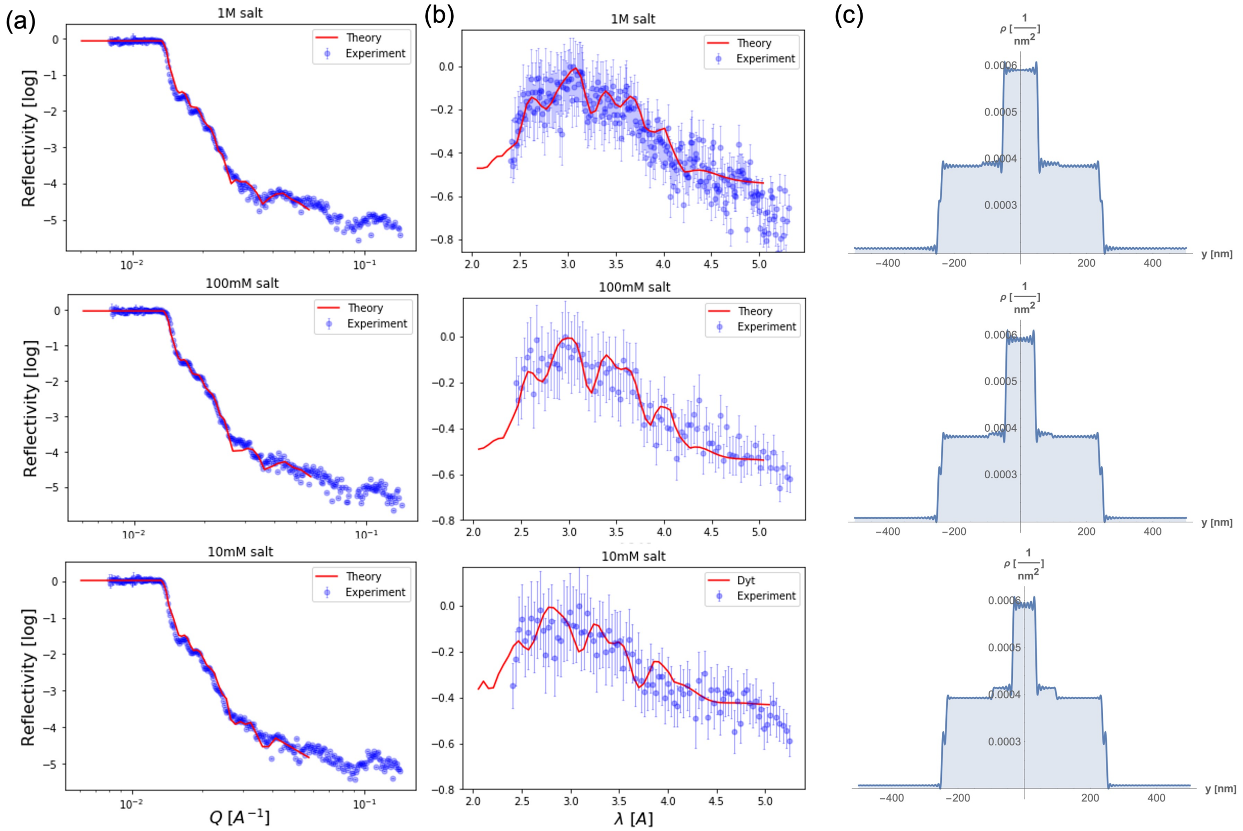

Figure S9: **Simultaneus fits to the specular and off-specular signals for sample in pH 4 and various salt concentrations; 10 mM, 100 mM, and 1M.** (a)Specular fits (b) off-specular fits, (c) SLD profile of the channel layer containing the polymer brushes. The plot shows a single channel (unit cell) such that $y = 0$ is set to be at the middle of a channel. The middle SLD with the highest value at the center of the channel represents the solvent or the gate between the polymer brushes on the opposite sidewalls of the channel. The two adjacent plateaus represent regions with different volume fractions of the polymer brush, indicating a relatively denser region near the sidewalls and a dilute region away from the sidewall.

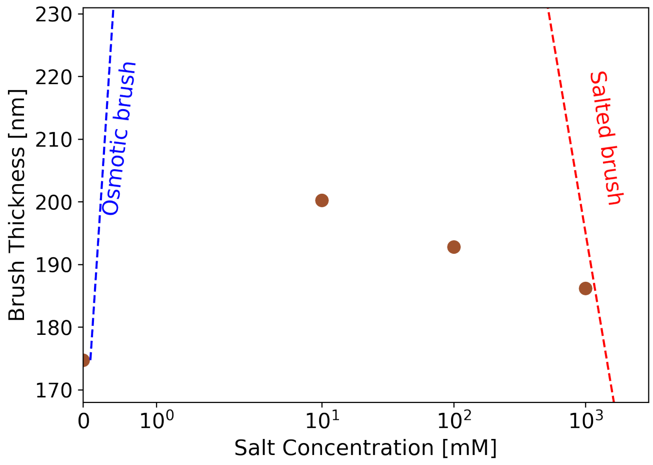

Figure S10: **Brush thickness as a function of salt concentration.** The dashed lines represent the classic scaling profile in the "polyelectrolyte" regime, where the influence of excluded volume is not considered.

References

(1) Morse, A. J.; Edmondson, S.; Dupin, D.; Armes, S. P.; Zhang, Z.; Leggett, G. J.; Thompson, R. L.; Lewis, A. Biocompatible polymer brushes grown from model quartz fibres: synthesis, characterisation and in situ determination of frictional coefficient. *Soft Matter* **2010**, *6*, 1571–1579.

(2) Ashkar, R.; Stonaha, P.; Washington, A. L.; Shah, V. R.; Fitzsimmons, M. R.; Maranville, B.; Majkrzak, C. F.; Lee, W. T.; Schaich, W. L.; Pynn, R. Dynamical theory calculations of spin-echo resolved grazing-incidence scattering from a diffraction grating. *J. Appl. Crystallogr.* **2010**, *43*, 455–465.

(3) Ashkar, R.; de Haan, V. O.; van Well, A. A.; Dalgliesh, R.; Plomp, J.; Fitzsimmons, M. R.; Schaich, W. L.; Pynn, R. Comparison of dynamical theory and phase-object approximation for neutron scattering from periodic structures. *Journal of Applied Crystallography* **2011**, *44*, 958–965.

(4) Ashkar, R.; Stonaha, P.; Washington, A. L.; Shah, V. R.; Fitzsimmons, M.; Maranville, B.; Majkrzak, C. F.; Lee, W.; Schaich, W.; Pynn, R. Dynamical theory calculations of spin-echo resolved grazin Pincidence scattering from a diffraction grating. *Journal of Applied Crystallography* **2010**, *43*, 455–465.

(5) Zhou, X.-L.; Chen, S.-H. Theoretical foundation of X-ray and neutron reflectometry. *Physics Reports* **1995**, *257*, 223–348.
